# Gastric vascular and motor responses to anaphylactic hypotension in anesthetized rats, in comparison to those with hemorrhagic or vasodilator-induced hypotension

**DOI:** 10.1007/s12576-017-0527-y

**Published:** 2017-01-31

**Authors:** Yuhichi Kuda, Toshishige Shibamoto, Tao Zhang, Wei Yang, Mamoru Tanida, Yasutaka Kurata

**Affiliations:** 1 Department of Physiology II, Kanazawa Medical University, Uchinada, Kahoku-gun, Ishikawa 920-0293 Japan; 2 Department of Colorectal and Hernia Surgery, The Fourth Affiliated Hospital of China Medical University, Shenyang, 110032 China; 3 Department of Infectious Disease, Shengjing Hospital of China Medical University, Shenyang, 110004 China

**Keywords:** Anaphylactic shock, Hemorrhage, Gastric circulation, Gastric motility, Gastric emptying, Anesthetized rats

## Abstract

Anaphylactic shock is life-threatening, but pathophysiology of the stomach lesion remains unclear. We determined gastric hemodynamics and gastric functions during anaphylactic hypotension, as compared to hypotension induced by hemorrhage or sodium nitroprusside (SNP) in anesthetized and ovalbumin-sensitized Sprague–Dawley rats. Systemic arterial pressure, portal venous pressure, and gastric arterial blood flow were measured, and gastric vascular resistance (GVR) was determined. Separately, the intragastric pressure (IGP) and gastric effluent, as a measure of gastric flux, were continuously measured. During anaphylaxis, GVR decreased only transiently at 0.5 min, followed by an increase. IGP increased markedly, while gastric flux decreased. During hemorrhage, GVR and IGP increased, while gastric flux did not change. When SNP was injected, both GVR and IGP decreased and gastric flux increased only just after injection. In conclusion, gastric vasodilatation occurs only transiently after antigen injection, and gastric motility increases, but gastric emptying deceases during anaphylactic hypotension in anesthetized rats.

## Introduction

Anaphylactic shock triggered by an allergic reaction is potentially life-threatening. Abdominal cramps and emesis are clinically observed in patients suffering from anaphylactic shock [1]. These symptoms may be related to a change of the gastric motility [2]. Previous articles have reported that the stomach strips are contracted by the chemical mediators released in response to anaphylactic reactions [3–5]. This evidence suggests that gastric motility is increased by anaphylactic chemical mediators. In contrast, it was reported that gastric luminal challenge with specific antigen decreased a phasic antral activity in sensitized rats [3]. On the other hand, gastric emptying was reported to be delayed when the gastric luminal challenge with specific antigen was performed in sensitized rats [3]. However, either gastric motility or gastric emptying has not been evaluated during anaphylactic hypotension. The primary aim of this study was therefore to determine the gastric motility and gastric emptying during anaphylactic shock by measuring continuously the intra-gastric pressure (IGP) and gastric effluent in anesthetized rats.

It is well known that anaphylactic hypotension is characterized by vasodilation, which is expected to increase blood flow to an affected region. Conversely, previous papers reported that gastric arterial blood flow decreased to 33 or 25% of baseline during anaphylactic shock in anesthetized rats [6, 7]. However, in these reports, blood flows were measured by the microsphere method, and therefore were not measured continuously. These intermittent measurements of blood flow might have missed the rapid and transient alterations that occurred during anaphylaxis. Indeed, we reported that blood flow of the mesenteric artery [8] and hepatic artery [9], portal vein [9], and femoral artery [10] increased at an early stage in the anaphylactic shock. Thus, a continuous blood flow measurement is required to observe quick changes precisely during anaphylactic shock, especially at the early stage, when blood pressure falls rapidly. Therefore, the second aim of this study was to determine the gastric vascular resistance during systemic anaphylactic hypotension by measuring continuously the gastric arterial blood flow with Doppler ultrasonic blood flow probes in anesthetized rats. In addition, in order to find clearly the characteristics of anaphylactic hypotension, we compared the gastric vascular and motor responses to anaphylactic hypotension with those to hemorrhage or vasodilator-induced hypotension, in which the blood pressure was decreased to a similar extent to anaphylactic hypotension.

## Materials and methods

### Animals

Fifty-six male Sprague–Dawley (SD) rats (Japan SLC, Shizuoka, Japan) weighing 350 ± 5 g were used in this study. Rats were maintained at 23 °C and under pathogen-free conditions on a 12:12-h dark/light cycle, and allowed food and water ad libitum. The experiments conducted in the present study were approved by the Animal Research Committee of Kanazawa Medical University (2015-48).

### Sensitization

Rats were sensitized by a subcutaneous injection of an emulsion made by mixing equal volumes of complete Freund’s adjuvant (0.5 ml) with 1 mg ovalbumin (grade V, sigma) dissolved in physiological saline (0.5 ml). Two weeks after injection, rats were used for the following experiments. Non-sensitized rats were injected with complete Freund’s adjuvant with saline [11].

### Surgical preparation

Following a 12-h fast with free access to water, the rats were anesthetized with pentobarbital sodium (50 mg/kg, i.p.) and placed supinely on a thermostatically controlled heating pad (ATC-101B, Unique Medical, Japan) that maintained body temperature at 36–37 °C throughout the experiment. The adequacy of anesthesia was monitored by the stability of blood pressure and respiration under control conditions and during a pinch of the hindpaw. Supplemental doses of the anesthetic (10% of the initial dose) were given intraperitoneally as necessary. The trachea was intubated to facilitate spontaneous breathing. The right carotid artery was catheterized with a polyethylene tube (ID 0.6 mm, OD 0.9 mm) for measurement of the systemic arterial pressure (SAP). The right jugular vein was catheterized with a polyethylene tube (ID 0.6 mm, OD 1.0 mm) for measurement of the central venous pressure (CVP) at expiration. The right femoral vein was catheterized with a polyethylene tube (ID 0.5 mm, OD 0.8 mm) for a continuous infusion of saline (10 ml/kg/h). In the hemorrhage group, the right femoral artery was catheterized with a polyethylene tube (ID 0.5 mm, OD 0.8 mm) for blood removal.

#### Experiment on gastric blood flow

After a midline incision of the abdomen, a pulsed Doppler flow probe (MC0.7PSB, Transonic Systems, Ithaca, NY, USA) was placed on the gastric artery for measurement of the mean gastric arterial blood flow (GABF). The portal vein was inserted with a 24-guage polyethylene catheter (Terumo, Tokyo, Japan) for measurement of the portal venous pressure (PVP).

Sensitized rats were assigned to the anaphylaxis group (*n* = 7), and the non-sensitized rats to the control group (*n* = 7), the hemorrhage group (*n* = 7), and the vasodilator sodium nitroprusside (SNP) group (*n* = 7). The SAP, CVP, and PVP were continuously measured with pressure transducers (TP-400T, Nihon-Kohden, Tokyo, Japan), and the reference level was set at the right atrium. Heart rate (HR) was measured by analyzing the data of SAP (PowerLab Systems, ML870, AD Instruments, Castle Hill, Australia).

Hemodynamic parameters were recorded for at least 30 min after surgery. After the baseline measurements, the ovalbumin antigen (0.6 mg, i.v.) was administered to rats of the anaphylaxis group and the control group. The SNP (3 mg/kg, s.c.) was administered to rats of the SNP group. In the hemorrhage group, bleeding started through the right femoral artery catheter with a syringe pre-rinsed with heparin at an appropriate speed to reduce SAP in a manner similar to that in the anaphylaxis group. The proper volume of blood was re-perfused if necessary. These hemodynamic variables were digitally displayed and recorded at 40 Hz by PowerLab. The gastric vascular resistance (GVR) was calculated as follows: GVR = (SAP−PVP)/GABF.

#### Experiment on gastric motility and emptying

After a midline incision of the abdomen, two polyethylene cannulas (ID 1.8 mm, OD 2.2 mm) combined with glue were introduced via the forestomach and positioned in the antrum; one for measurement of the IGP and another for injection of warmed saline [12]. The infusion cannula was connected to a Marriotte bottle filled with warm saline to perfuse the stomach at constant IGP, which was maintained at 0–2 mmHg during baseline by adjusting the height of the Marriotte bottle [12, 13]. Once the height of the bottle was settled, it was maintained throughout the experimental period. The esophagus was ligated at the esophagogastric junction. An effluent polyethylene cannula (ID 4.0 mm, OD 3.6 mm) was placed in the duodenum but not introduced into the pylorus. The gastric effluent (GE) was collected drop by drop in a tube suspended from the force transducer (SB-1T, Nihon-Kohden) and its weight was cumulatively measured for determining the gastric flux rate or gastric emptying. The four groups including the anaphylaxis group (*n* = 7), control group (*n* = 7), hemorrhage group (*n* = 7), and SNP group (*n* = 7) were studied in the same manner as that in the experiment on gastric blood flow.

### Statistics

All results are expressed as the mean ± SEM. Statistical analyses were performed with repeated-measures analysis of variance, and a *p* value less than 0.05 was considered significant. When a significant difference was obtained, post hoc analysis was performed by the Dunnett’s test. Comparison of individual data among the four groups was performed by analysis of variance followed by the Tukey test. A *p* value less than 0.05 was considered significant.

## Results

### Experiment on gastric blood flow

**Fig. 1:**
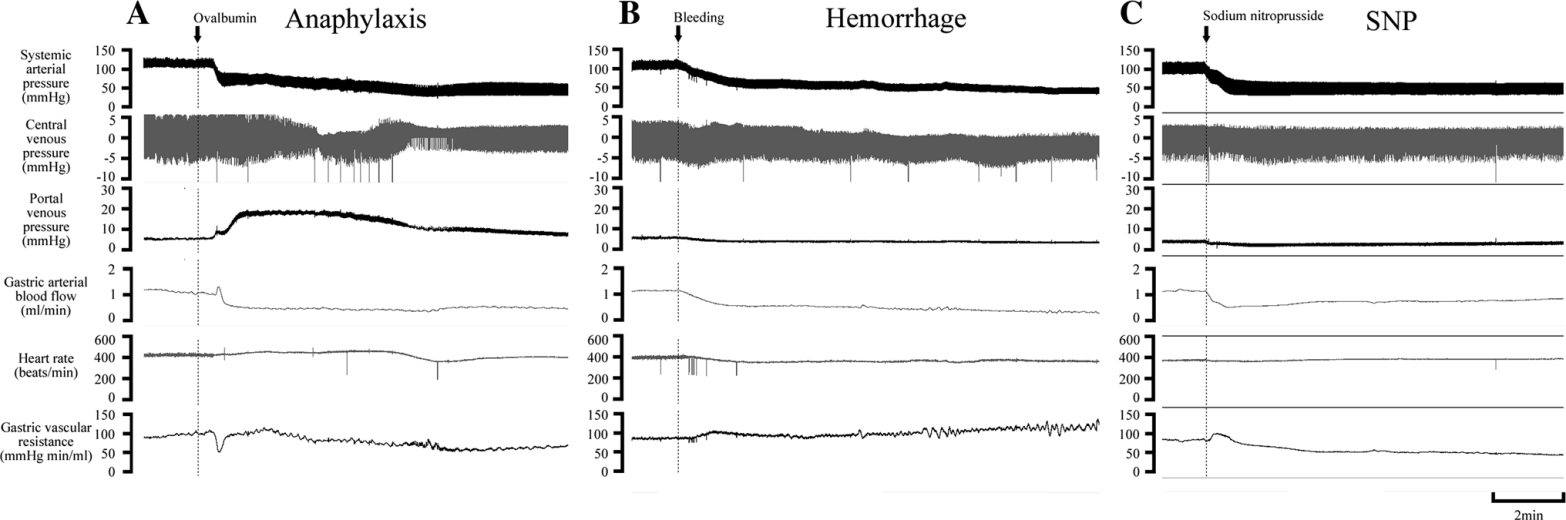
Representative recordings of the systemic arterial pressure, central venous pressure, portal venous pressure, gastric arterial blood flow, heart rate and gastric vascular resistance in the anaphylaxis (**a**), hemorrhage (**b**), and sodium nitroprusside (SNP) (**c**) groups

**Fig. 2:**
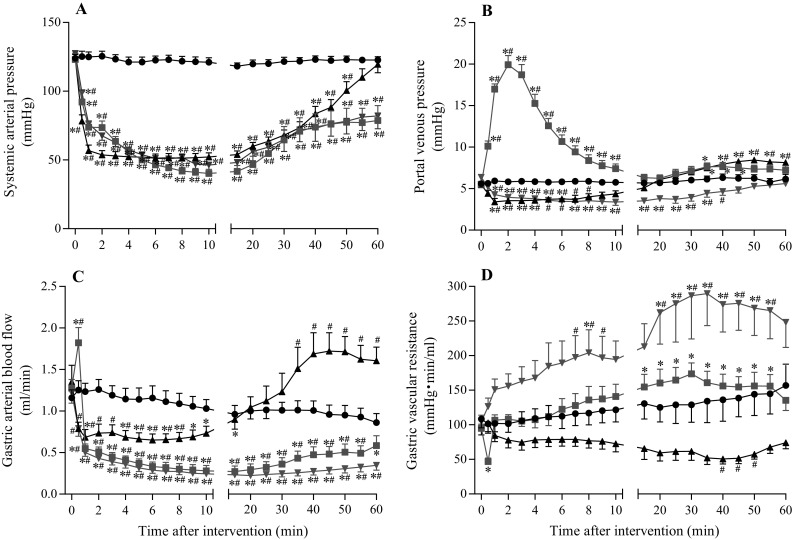
Time-course data of systemic arterial pressure (**a**), portal venous pressure (**b**), gastric arterial blood flow (**c**), and gastric vascular resistance (**d**), after the start of the interventions. *Circle*, the control group (*n* = 7); *square*, the anaphylaxis group (*n* = 7); *inverted triangle*, the hemorrhage group (*n* = 7); *triangle*, the SNP group (*n* = 7). Values are mean ± SEM; *p < 0.05 vs. baseline; ^#^ *p* < 0.05 vs. the control group

Figure 1a shows a representative example of hemodynamic changes in the anaphylaxis group. The time course changes in SAP, PVP, GABF, and GVR of all groups are summarized in Fig. 2 (square, the anaphylaxis group; inverted triangle, the hemorrhage group; triangle, the SNP group; circle, the control group). As shown in Fig. 1a, in the anaphylaxis group, SAP decreased significantly from the baseline of 123 ± 4 to 73 ± 4 mmHg at 1 min after antigen administration, reached a nadir of 40 ± 2 mmHg at 10 min, and then gradually returned to 79 ± 10 mmHg at 60 min. PVP increased from the baseline of 5.5 ± 0.2 mmHg to the peak of 19.9 ± 1.1 mmHg at 2 min after antigen administration and returned to the baseline level. GABF increased from the baseline of 1.3 ± 0.1 to 1.8 ± 0.2 ml/min at 0.5 min and then decreased progressively to a nadir of 0.3 ± 0.1 ml/min (23% of baseline) at 15 min after antigen injection, followed by a gradual return to 0.6 ± 0.1 ml/min at 60 min. Consequently, GVR decreased from the baseline of 95 ± 6 to 47 ± 5 mmHg min/ml at 0.5 min after antigen injection, returned to the baseline level at 1 min, and then did not change significantly until 10 min. Thereafter, GVR remained elevated at the level of around 135% baseline towards the end of the experiment.

Figure 1b shows a representative example of hemodynamic changes in a rat suffering from hemorrhage in which blood was withdrawn so that SAP decreased in the same manner as observed in the anaphylaxis group. The maximal volume of blood shed was 6.6 ± 0.4 ml, and the blood loss was reinfused in the late phase. In contrast to the results from the anaphylaxis group, PVP significantly decreased from the baseline of 6.4 ± 0.2 to 4.2 ± 0.3 mmHg at 1 min after hemorrhage, and then it remained decreased until 35 min (Fig. 2b). GABF decreased similarly to that of the anaphylaxis group (Fig. 2c). Consequently GVR increased after hemorrhage, which was significantly higher than that in the anaphylaxis group (Fig. 2d).

Figure 1c shows a representative example of the hemodynamic response to SNP. SAP decreased more rapidly than that of the anaphylaxis group, and then it showed a similar change to that in the anaphylaxis group. PVP decreased similarly to that of the hemorrhage group until 10 min (Fig. 2b). GABF decreased significantly at 0.5 min, and recovered to the baseline at 20 min (Fig. 2c). GVR tended to decrease, but not significantly, from baseline after injection (Fig. 2d). However, the GVR levels at 40–50 min after SNP injection were significantly smaller than that in the control group (Fig. 2d).

### Experiment on gastric motility and emptying

**Fig. 3:**
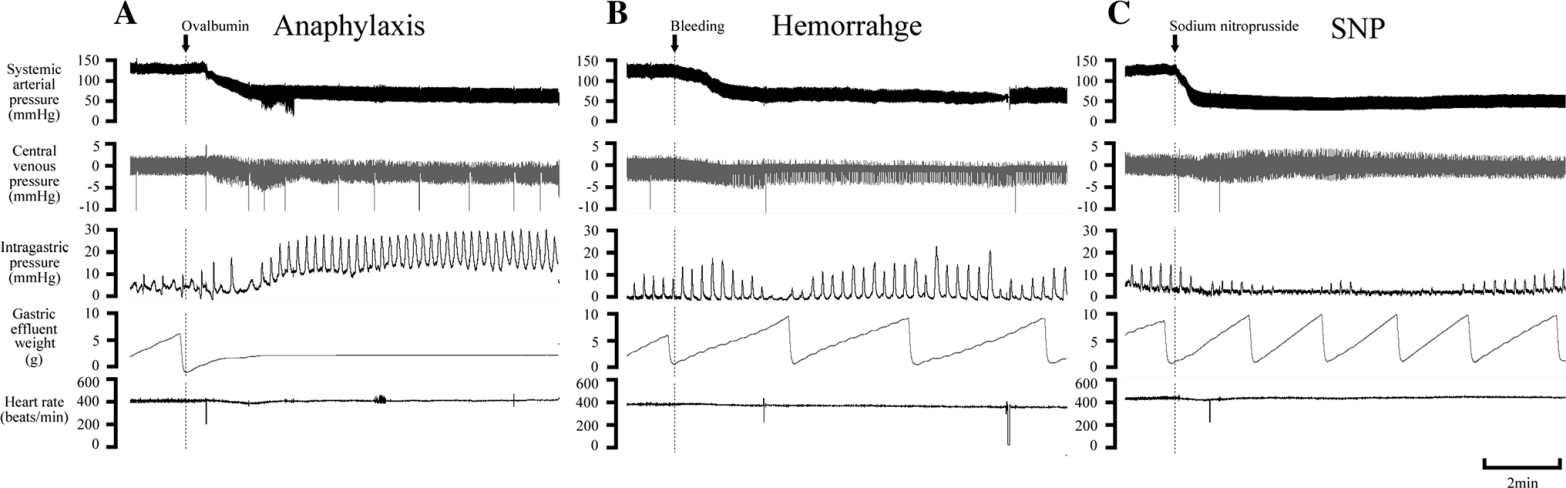
Representative recordings of the systemic arterial pressure, central venous pressure, intragastric pressure, gastric effluent weight, and heart rate in the anaphylaxis (**a**), hemorrhage (**b**), and SNP (**c**) groups

**Fig. 4:**
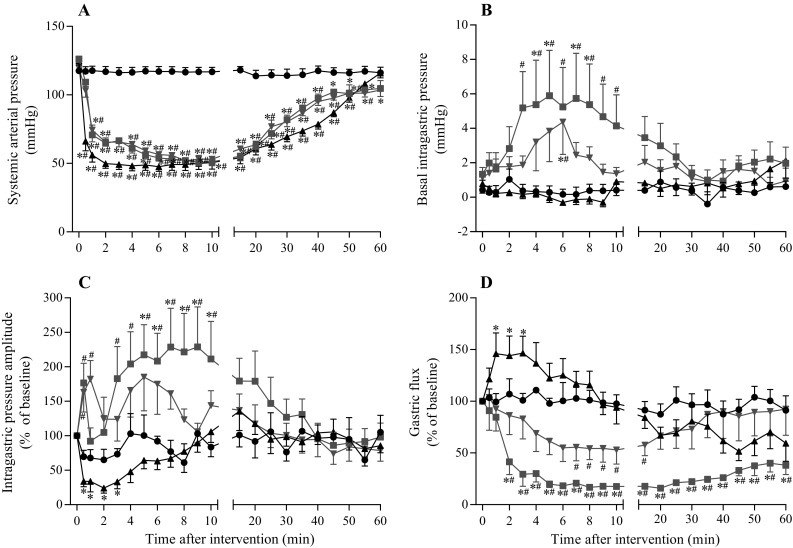
Time-course data of systemic arterial pressure (**a**), basal intragastric pressure (**b**), intragastric pressure amplitude (**c**), and gastric flux (**d**) in the anaphylaxis, hemorrhage, and SNP groups. *Circle*, the control group (*n* = 7); *square*, the anaphylaxis group (*n* = 7); *inverted triangle*, the hemorrhage group (*n* = 7); *triangle*, the SNP group (*n* = 7). Values are mean ± SEM; *p < 0.05 vs. baseline; ^#^ *p* < 0.05 vs. the control group

Figure 3 shows a representative example of the responses of IGP and gastric flux in the three hypotension groups. The time-course changes in the variables of all groups are summarized in Fig. 4. In all hypotension groups, SAP changed similarly, as observed in the experiment on gastric blood flow, as described above.

In the anaphylaxis group, as shown in Fig. 3a, at 1 min after antigen injection the gastric flux was stopped and thereafter did not return to the baseline, but remained at the low level of 38% at 60 min after antigen (Fig. 4d). The basal IGP significantly increased from the baseline of 1.3 ± 0.4 to 5.9 ± 2.6 mmHg and the IGP amplitude also increased by 117 ± 44% from the baseline of 7.9 ± 1.7 mmHg at 5 min when the gastric flux was almost stopped, a finding indicating an increase in gastric contractility (Fig. 4b,c). In the hemorrhage group, the basal IGP increased only slightly from the baseline of 1.1 ± 0.3 to 4.4 ± 1.9 mmHg at 6 min (Fig. 4b). The IGP amplitude tended to increase, but not significantly, after hemorrhage (Fig. 4c). In the SNP group, the basal IGP did not change significantly (Fig. 4b), while the IGP amplitude decreased by 67 ± 8% from the baseline of 8.7 ± 1.7 mmHg at 0.5 min after SNP administration, and remained decreased until 3 min (Fig. 4c).

Gastric flux in the anaphylaxis group significantly decreased from the baseline of 3.4 ± 0.4 to 1.3 ± 0.4 mg/min (41 ± 12% of the baseline) at 2 min, and thereafter remained at low level less than 15% of the baseline. Gastric flux in the hemorrhage group decreased slightly but not significantly. Gastric flux in the SNP group increased significantly only just after SNP injection (1–3 min), and then soon returned to the baseline. The percent changes in gastric flux are shown in Fig. 4d.

## Discussion

We here determined the responses of the GVR, basal gastric motility and gastric flux to anaphylactic hypotension, in comparison to those to the hemorrhagic and vasodilator-induced hypotension, in anesthetized rats. There are three major findings. First, in the anaphylaxis group, immediately after antigen administration, GVR decreased only transiently, along with GABF elevation. Thereafter, it did not increase significantly until 10 min after antigen. In contrast, GVR increased during hemorrhagic hypotension, while it decreased during SNP-induced hypotension. Second, both the basal IGP and the peak amplitude of IGP were significantly increased in the anaphylaxis group, while they increased only slightly and decreased significantly in the hemorrhage and SNP groups, respectively. Finally, gastric emptying, as evaluated by gastric flux, was markedly depressed in the anaphylaxis group, while it increased only temporarily in the SNP group. It did not change significantly in the hemorrhage group.

To the best of our knowledge, this is the first study to determine the GVR in anaphylactic shock models. We clearly showed that vasodilatation of the gastric vascular beds definitely occurred transiently only in the early phase of anaphylaxis. This is consistent with the initial and transient decrease in total peripheral resistance during anaphylactic shock in rats [8]. We also previously showed that vasodilatation of the hepatic artery and splanchnic artery occurred in the early phase of anaphylaxis [9, 14]. It should also be noted that the transient increase in GABF coincided with the start of fall in SAP. This suggests that vasodilatation of these arteries seems to trigger the fall of SAP. In this respect, the blood flow of the common carotid artery with the occlusion of the distal lateral carotid artery, which mainly supplies the brain, did not increase, suggesting absence of vasodilatation, in the same rat anaphylactic hypotension model [15]. Regional differences in the vasomotor responses to anaphylaxis may exist, and further study will be required.

In contrast, GVR in the hemorrhage group increased markedly throughout the experiment period. The magnitude of the GVR increase in the present study was comparable to that of similar hemorrhage in the rat [16]. Enhanced gastric vasoconstriction during hemorrhagic hypotension seems to be a compensatory response for redistribution of blood flow to the brain and heart under the hypovolemic state. This contrasts with the only modest GVR increase in the late phase in the anaphylaxis group (Fig. 2d), suggesting the absence or weakness of the compensatory response during anaphylactic hypotension. The reasons for the difference in the GVR response between the anaphylaxis and hemorrhage groups are currently not known. In the face of systemic hypotension, the arterial baroreceptor reflex should activate the sympathetic nervous system, resulting in gastric vasoconstriction. This is the case of the hemorrhage group. In contrast, in the anaphylaxis group, arterial vasodilatation of the gastric vascular beds, which could be caused by chemical mediators released in the anaphylactic reactions, might counteract baroreceptor reflex-mediated vasoconstriction. Indeed, the pentobarbital anesthetized rats are weak in the sympatho-excitatory response to anaphylactic hypotension [17]. Furthermore, baroreflex has little augmenting effects on the sympathetic response in anaphylaxis [17]. With respect to the mechanism for the increased GVR at late phase of anaphylactic hypotension, it could be caused by increased sympathetic nerve activity, albeit a small magnitude [17]. Another one may be related to increased vascular permeability: administration of antigen to sensitized rats produces an increase in vascular permeability to plasma proteins in the stomach [18], which undoubtedly induces an efflux of water into the extravascular space with a resultant increase in interstitial water around the gastric vessels, leading to an apparent decrease in GABF, and consequently an increase in GVR.

In the present study, we continuously measured the IGP. Here we have shown that anaphylactic hypotension causes an increase in gastric motility, as evidenced by increases in basal levels and amplitude of IGP. Of note, increased gastric motility was observed in the present anaphylaxis rats under anesthesia of pentobarbital, which could suppress gastric motility in the rat [19]. However, Catto-Smith et al. [3] reported that gastric motility transiently decreased in response to the gastric luminal challenge with specific antigen. In their study, the anaphylactic reaction was induced only in the gastric region. Furthermore, they did not measure the blood pressure. Therefore, it is not clear whether hypotension occurred or not in the rats treated with the gastric antigen. In the current study, we used the systemic anaphylactic shock model, which was different from the regionally induced gastric anaphylaxis model of Catto-Smith et al. [3]. Moreover, the same authors reported that the contractility of the isolated stomach strips derived from the ovalbumin-sensitized rats increased in response to the antigen challenge. This finding is consistent with our results.

The mechanism for the increased gastric motility during the anaphylactic shock remains unclear. Three possibilities could be considered for the increase in gastric motility: it may be related to (1) the blood pressure decrease, (2) the changes in the autonomic nervous system activity and (3) the effects of released chemical mediators. Concerning the blood pressure decrease, in the present study gastric motility increased during the anaphylactic hypotension and hemorrhagic hypotension, but decreased during the SNP-induced hypotension. A previous study reported that the gastric motility was decreased by the hypobaric hypoxia-induced hypotension [20]. Therefore, these results indicate that the gastric motility did not necessarily depend on a change in the blood pressure. Second, in respect to effects of the autonomic nervous system, it is well known that activation of the splanchnic nerves, the sympathetic nerves innervating the stomach, inhibit gastric contraction [21, 22], while the vagal nerves facilitate gastric contraction via the muscarinic receptors [21, 22]. We reported that anaphylactic hypotension increased the renal sympathetic nerve activity [17], but did not significantly change the hepatic sympathetic nerve activity [23]. However, it remains unknown whether the gastric sympathetic nerve activity increases or not. Moreover, the response of the vagal nerve activity to anaphylactic shock also remains unclear. Finally, concerning the chemical mediators, it was reported that the antigen-induced contraction was significantly reduced by the mast cell stabilizer [3], which indicates that chemical mediators released from mast cells have a facilitative effect on gastric motility during the anaphylactic shock. Actually, stomach strips are contracted by anaphylaxis-associated mediators such as histamine [3], serotonin [3, 4], LTD_4_ [3, 5], and platelet-activating factor [3, 24].

In the present study, the gastric excretion deceased during the anaphylactic shock. This finding is consistent with the report of Catto-Smith et al. [25]: regional gastric anaphylaxis delayed gastric emptying in anesthetized rats. They showed that the gastric emptying rate of water labeled with radioisotope was significantly smaller in the sensitized rats than in the sham non-sensitized rats at 20 min after intra-gastric administration of ovalbumin antigen. The present study reinforces this finding by the continuous measurement of the gastric effluent. About the mechanism for the deceased gastric emptying during anaphylactic hypotension, we assume that gastric excretion is decreased by pylorus resistance because the gastric motility was increased, as evidenced by the increase in IGP. Indeed, pylorus was contracted by histamine [26, 27] and serotonin [28]. The antral pump and pyloric opening are important factors for the gastric emptying [29], and their dysfunctions may occur during the anaphylactic shock. Increased gastric motility in the presence of the pylorus contraction may account for the abdominal cramps and emesis, as observed in patients suffering from systemic anaphylaxis [1]. In addition, delayed gastric emptying demonstrated in the present study may also explain in part the mechanism for vomiting in patients with systemic anaphylaxis [1], because delayed gastric emptying is associated with the occurrence of vomiting [30].

In the present study, in contrast to the anaphylactic hypotension, gastric emptying did not change during hemorrhage. However, Gondium et al. [31] reported that liquid gastric emptying was increased by 28.5–49.9% in response to hypotension induced by controlled bleeding (1.5 ml/100 g) in awake rats. The difference in results between this study and ours is not known, but three explanations could be provided: (1) awake rats vs. anesthetized rats; (2) shed blood volume, low (1.5 ml/100 g) vs. high (1.9 ml/100 g); and (3) Wistar rats (180–200 g) vs. SD rats (345–355 g). On the other hand, in response to a subcutaneous injection of SNP in the present study, the gastric excretion increased only transiently just after SNP injection in the presence of decreased gastric motility. This transient increase in gastric flux may be caused by SNP-induced sphincter muscle relaxation of the pylorus [32], which could reduce gastroduodenal resistance, resulting in increased effluent from the stomach perfused at the constant pressure by the Marriotte bottle.

In conclusion, the systemic anaphylaxis in rats is characterized by transient vasodilation of gastric vascular beds at the beginning of hypotension. Anaphylactic hypotension is accompanied by increased gastric motility, but decreased gastric emptying.
